# Ergodic Stationary Distribution of a Stochastic Hepatitis B Epidemic Model with Interval-Valued Parameters and Compensated Poisson Process

**DOI:** 10.1155/2020/9676501

**Published:** 2020-01-22

**Authors:** Driss Kiouach, Yassine Sabbar

**Affiliations:** ^1^Modelling, Systems and Technologies of Information Team, High School of Technology, Ibn Zohr University, Agadir, Morocco; ^2^National School of Applied Sciences, Ibn Zohr University, Agadir, Morocco

## Abstract

Hepatitis B epidemic was and is still a rich subject that sparks the interest of epidemiological researchers. The dynamics of this epidemic is often modeled by a system with constant parameters. In reality, the parameters associated with the Hepatitis B model are not certain, but the interval in which it belongs to can readily be determined. Our paper focuses on an imprecise Hepatitis B model perturbed by Lévy noise due to unexpected environmental disturbances. This model has a global positive solution. Under an appropriate assumption, we prove the existence of a unique ergodic stationary distribution by using the mutually exclusive possibilities lemma demonstrated by Stettner in 1986. Our main effort is to establish an almost perfect condition for the existence of the stationary distribution. Numerical simulations are introduced to illustrate the analytical results.

## 1. Introduction

Hepatitis B is an enormous defiance and a great global health issue caused by the Hepatitis B virus (HBV) [2]. Chronic HBV can be transmitted by sexual contact, through the touch, by impregnation with polluted blood, or by the direct transmission of Hepatitis B from the mother to a fetus during pregnancy (vertical transmission) [3]. According to the recent statistics of world health organization (WHO) [4], about 350 million people worldwide have been infected and carrying HBV. This serious infection is responsible for approximately 600,000 deaths each year [5]. Because of the high severity of HBV infection and a large number of deaths associated with it, it is compulsory to improve our control of this virus. Mathematical models are a vigorous tool to simulate and control the spread of the HBV infection. There exist many previous interesting works committed to studying Hepatitis B transmission. For example, Anderson and May [6] analyzed a straightforward mathematical model for illustrating the role of carrier individuals on the spread of HBV. In [7, 8], the authors developed the impact of vaccination and other controlling measures of HBV outbreak. They showed that the booster vaccine of Hepatitis B is very necessary and useful. Khan et al. [9] formulated the characteristics of HBV disease transmission and proposed the following deterministic Susceptible (*S*)—Infected (*I*)—Recovered (*R*) model:

(1) $$\begin{array}{c} \left\{\begin{array}{c} \dot{S}\left(t\right)=A-\beta S\left(t\right)I\left(t\right)-\left(\mu +\theta \right)S\left(t\right),\\ \dot{I}\left(t\right)=\beta S\left(t\right)I\left(t\right)-\left(\mu +\delta +r\right)I\left(t\right),\\ \dot{R}\left(t\right)=\delta I\left(t\right)+\theta S\left(t\right)-\mu R\left(t\right), \end{array}\right. \end{array}$$

with initial data *S*(0)=*S*_0_ > 0, *I*(0)=*I*_0_ > 0, and *R*(0)=*R*_0_ > 0. The positive parameters of the deterministic model (1) are given in the following list. The deterministic model constructed above can be improved by taking into account the unpredictable biological conditions [10–14]. Also, environmental fluctuations have important effects on the growth and propagation of an epidemic disease [15, 16]. Khan et al. [9] discussed the dynamics of a stochastic Hepatitis B epidemic model with varying population size. They supposed that the effect of the random fluctuations is manifested as a perturbation in the Hepatitis B transmission rate. To confer the realistic aspect to our study and make it biologically reasonable, in this study, we extend the work of Khan et al. [9] to the case of Lévy noise perturbation. We take into consideration the effects due to some unexpected and severe environmental disturbances (tsunami, floods, earthquakes, hurricanes, whirlwinds, etc.) on the disease outbreak [17, 18]. Thus, we consider the following model:

(2) $$\begin{array}{c} \left\{\begin{array}{c} \text{d}S\left(t\right)=\left(A-\beta S\left(t\right)I\left(t\right)-\left(\mu +\theta \right)S\left(t\right)\right)\text{d}t-\sigma S\left(t^{-}\right)I\left(t^{-}\right)\text{d}W\left(t\right)-\int_{Z}\eta \left(u\right)S\left(t^{-}\right)I\left(t^{-}\right)\tilde{N}\left(\text{d}t,\text{d}u\right),\\ \text{d}I\left(t\right)=\left(\beta S\left(t\right)I\left(t\right)-\left(\mu +\delta +r\right)I\left(t\right)\right)\text{d}t+\sigma S\left(t^{-}\right)I\left(t^{-}\right)\text{d}W\left(t\right)+\int_{Z}\eta \left(u\right)S\left(t^{-}\right)I\left(t^{-}\right)\tilde{N}\left(\text{d}t,\text{d}u\right),\\ \text{d}R\left(t\right)=\left(\delta I\left(t\right)+\theta S\left(t\right)-\mu R\left(t\right)\right)\text{d}t, \end{array}\right. \end{array}$$

where *S*(*t*^−^) and *I*(*t*^−^) are the left limits of *S*(*t*) and *I*(*t*), respectively. *W*(*t*) is a real-valued Brownian motion with intensity *σ* > 0 defined on a complet probablity space (*Ω*, *ℱ*, *ℙ*) with a filtration {*ℱ*_*t*_}_*t*≥0_ satisfying the usual conditions. *N* is a Poisson counting measure with the compensator $\tilde{N}$ and the characteristic measure ν on a measurable subset *Z* of (0, *∞*) satisfying *ν*(*Z*) < *∞*. *W*(*t*) is independent of *N*. We assume that ν is a Lévy measure such that $\tilde{N}\left(\text{d}t,\text{d}u\right)=N\left(\text{d}t,\text{d}u\right)-\nu \left(\text{d}u\right)\text{d}t$. The bounded function *η* :  *Z* × *Ω*⟶*ℝ* is *ℬ*(*Z*) × *ℱ*_*t*_-measurable and continuous with respect to ν.

In system (2), we assume that model parameters (see Table 1) are precisely known and constant. However, this hypothesis may not be validated due to the lack of data and errors of measurements. It is more realistic to study Hepatitis B dynamics with interval-valued parameters. Recently, Pal et al. [19] used interval-valued parameters to analyze the prey-predator model due to the lack of precise biological data such as prey and predator population growth rates. The same logic was applied for epidemic models. In [20], the authors treated a cholera epidemic model with uncertain parameters. They investigated the stability condition of equilibrium points. Bao et al. [21] studied a stochastic SIRS model that includes Lévy jumps and interval parameters. They established the stochastic threshold which determines the extinction and persistence in the mean of disease. In [22], the authors studied an imprecise SIR epidemic model. They solved the optimal control problem.

In this paper, we consider the Hepatitis B epidemic model with stochastic transmissions and Lévy noise. To make our model more realistic, we consider imprecise biological parameters. To the best of our knowledge, the existence of a stationary distribution of system (2) with imprecise parameters remains not proved. In the next section, we propose a solution to the mentioned problem by considering an original method different from the Lyapunov approach described in [23]. Before proving the existence of unique a stationary distribution in Subsection 2.3, we demonstrate the well-posedness of the model (2) with interval-valued parameters in Subsection 2.2. Simulation examples are proposed in Subsection 2.4 to illustrate our theoretical study.

## 2. Main Results

### 2.1. Imprecise Stochastic Hepatitis B Model

Before showing the main result of this paper, we first present some definitions of interval numbers and interval-valued functions which are used in our study. Then, we construct the imprecise stochastic Hepatitis B model.

**Definition 1 (see [19]).**

An interval number *Z* is defined as $Z=\left[\overset{ˇ}{z},\hat{z}\right]=\left\{\left.x\right|\overset{ˇ}{z}\leq x\leq \hat{z},x\in \mathbb{R}\right\}$ where *ℝ* is the set of all real numbers and $\overset{ˇ}{z}$ and $\hat{z}$ are the lower and upper limits of the interval numbers, respectively. Furthermore, any real number *z* can be represented in terms of interval number as [*z*, *z*].

**Definition 2 (see [19]).**

An interval-valued function for the interval [*x*, *y*] can be represented by the following function:

(3) $$\begin{array}{c} \psi \left(p\right)=x^{\left(1-p\right)}y^{p},\;\text{for }p\in \left[0,1\right]. \end{array}$$

**Theorem 1 .**

The following stochastic differential equation with interval-valued parameters

(4) $$\begin{array}{c} \left\{\begin{array}{c} \text{d}S\left(t\right)=\left(\bar{A}-\bar{\beta }S\left(t\right)I\left(t\right)-\left(\bar{\mu }+\bar{\theta }\right)S\left(t\right)\right)\text{d}t-\bar{\sigma }S\left(t^{-}\right)I\left(t^{-}\right)\text{d}W\left(t\right)-\int_{Z}\eta \left(u\right)S\left(t^{-}\right)I\left(t^{-}\right)\tilde{N}\left(\text{d}t,\text{d}u\right),\\ \text{d}I\left(t\right)=\left(\bar{\beta }S\left(t\right)I\left(t\right)-\left(\bar{\mu }+\bar{\delta }+\bar{r}\right)I\left(t\right)\right)\text{d}t+\bar{\sigma }S\left(t^{-}\right)I\left(t^{-}\right)\text{d}W\left(t\right)+\int_{Z}\eta \left(u\right)S\left(t^{-}\right)I\left(t^{-}\right)\tilde{N}\left(\text{d}t,\text{d}u\right),\\ \text{d}R\left(t\right)=\left(\bar{\delta }I\left(t\right)+\bar{\theta }S\left(t\right)-\bar{\mu }R\left(t\right)\right)\text{d}t, \end{array}\right. \end{array}$$

where $\bar{A}\in \left[\overset{ˇ}{A},\hat{A}\right]$, $\bar{\beta }\in \left[\overset{ˇ}{\beta },\hat{\beta }\right]$, $\bar{\mu }\in \left[\overset{ˇ}{\mu },\hat{\mu }\right]$, $\bar{\theta }\in \left[\overset{ˇ}{\theta },\hat{\theta }\right]$, $\bar{\delta }\in \left[\overset{ˇ}{\delta },\hat{\delta }\right]$, $\bar{r}\in \left[\overset{ˇ}{r},\hat{r}\right]$ and $\bar{\sigma }\in \left[\overset{ˇ}{\sigma },\hat{\sigma }\right]$, is provided an interval-valued functional form of parameters by the following stochastic differential equation (SDE):

(5) $$\begin{array}{c} \left\{\begin{array}{c} \text{d}S\left(t\right)=\left({\left(\hat{A}\right)}^{1-p}{\left(\overset{ˇ}{A}\right)}^{p}-{\left(\hat{\beta }\right)}^{1-p}{\left(\overset{ˇ}{\beta }\right)}^{p}S\left(t\right)I\left(t\right)-\left({\left(\hat{\mu }\right)}^{1-p}{\left(\overset{ˇ}{\mu }\right)}^{p}+{\left(\hat{\theta }\right)}^{1-p}{\left(\overset{ˇ}{\theta }\right)}^{p}\right)S\left(t\right)\right)\text{d}t\\ -{\left(\hat{\sigma }\right)}^{1-p}{\left(\overset{ˇ}{\sigma }\right)}^{p}S\left(t^{-}\right)I\left(t^{-}\right)\text{d}W\left(t\right)-\int_{Z}\eta \left(u\right)S\left(t^{-}\right)I\left(t^{-}\right)\tilde{N}\left(\text{d}t,\text{d}u\right),\\ \text{d}I\left(t\right)=\left({\left(\hat{\beta }\right)}^{1-p}{\left(\overset{ˇ}{\beta }\right)}^{p}S\left(t\right)I\left(t\right)-\left({\left(\hat{\mu }\right)}^{1-p}{\left(\overset{ˇ}{\mu }\right)}^{p}+{\left(\hat{\delta }\right)}^{1-p}{\left(\overset{ˇ}{\delta }\right)}^{p}+{\left(\hat{r}\right)}^{1-p}{\left(\overset{ˇ}{r}\right)}^{p}\right)I\left(t\right)\right)\text{d}t\\ +{\left(\hat{\sigma }\right)}^{1-p}{\left(\overset{ˇ}{\sigma }\right)}^{p}S\left(t^{-}\right)I\left(t^{-}\right)\text{d}W\left(t\right)+\int_{Z}\eta \left(u\right)S\left(t^{-}\right)I\left(t^{-}\right)\tilde{N}\left(\text{d}t,\text{d}u\right),\\ \text{d}R\left(t\right)=\left({\left(\hat{\delta }\right)}^{1-p}{\left(\overset{ˇ}{\delta }\right)}^{p}I\left(t\right)+{\left(\hat{\theta }\right)}^{1-p}{\left(\overset{ˇ}{\theta }\right)}^{p}S\left(t\right)-{\left(\hat{\mu }\right)}^{1-p}{\left(\overset{ˇ}{\mu }\right)}^{p}R\left(t\right)\right)\text{d}t, \end{array}\right. \end{array}$$

for *p* ∈ [0,1].

The proof is similar to that in [19] and hence is omitted.

### 2.2. Well-Posedness of the Stochastic Model (5)

To investigate the dynamical behavior of the model (5), the first concerning thing is whether the model is well-posed.  Theorem 2 is a prerequisite for analyzing the long-run behavior of the model (5). From epidemiological considerations, it is reasonable to suppose that the intensity of Lévy jumps cannot exceed environmental carrying capacity. Hence, we impose the following standard assumption:

**Assumption 1 .**

The function *η*(*u*) is bounded and $\left|\left({\left(\hat{A}\right)}^{1-p}{\left(\overset{ˇ}{A}\right)}^{p}/{\left(\hat{\mu }\right)}^{1-p}{\left(\overset{ˇ}{\mu }\right)}^{p}\right)\eta \left(u\right)\right|\leq \Gamma <1$, *u* ∈ *Z*.

By using the Lyapunov analysis method (as mentioned in [21]), we shall verify that the solution of system (5) is global and positive.

**Theorem 2 .**

For any initial value (*S*_0_, *I*_0_, *R*_0_) ∈ *ℝ*_+_^3^, there exists a unique positive solution (*S*(*t*), *I*(*t*), *R*(*t*)) of system (5) on *t* ≥ 0, and the solution will remain in *ℝ*_+_^3^ with probability one. That is to say, the solution (*S*(*t*), *I*(*t*), *R*(*t*)) ∈ *ℝ*_+_^3^ for all *t* ≥ 0 almost surely.

**Proof**

Since the coefficients of system (5) satisfy the local Lipschitz condition, then for any initial value (*S*_0_, *I*_0_, *R*_0_) ∈ *ℝ*_+_^3^, there is a unique local solution (*S*(*t*), *I*(*t*), *R*(*t*)) on [0, *τ*_*e*_), where *τ*_*e*_ is the explosion time. To show that the solution is global, we only need to prove that *τ*_*e*_=*∞* a.s. Let *ϵ*_0_ > 0 be sufficiently large such that *S*_0_, *I*_0_, *R*_0_ lying within the interval [1/*ϵ*_0_, *ϵ*_0_]. For each integer *ϵ* ≥ *ϵ*_0_, we define the following stopping time:

(6) $$\begin{array}{c} \tau_{\epsilon }=\text{inf}\left\{t\in \left[0,\tau_{e}\right):\text{min}\left\{S\left(t\right),I\left(t\right),R\left(t\right)\right\}\leq \frac{1}{\epsilon }\text{ or max}\left\{S\left(t\right),I\left(t\right),R\left(t\right)\right\}\geq \epsilon \right\}, \end{array}$$

where, throughout this paper, we set inf∅=*∞* (as usual, ∅ denotes the empty set). Clearly, *τ*_*ϵ*_ is increasing as *ϵ*⟶*∞*. Set *τ*_*∞*_=lim_*ϵ*⟶*∞*_*τ*_*ϵ*_ whence *τ*_*∞*_ ≤ *τ*_*e*_. If we can prove that *τ*_*∞*_=*∞* a.s., then *τ*_*e*_=*∞* and the solution (*S*(*t*), *I*(*t*), *R*(*t*)) ∈ *ℝ*_+_^3^ for all *t* ≥ 0 almost surely. Specifically, to complete the proof, all we need is only to prove that *τ*_*∞*_=*∞* a.s. If this statement is false, then there exists a pair of positive constants *T* > 0 and *k* ∈ (0,1) such that

(7) $$\begin{array}{c} \mathbb{P}\left\{\tau_{\infty }\leq T\right\}>k. \end{array}$$

Hence, there is an integer *ϵ*_1_ ≥ *ϵ*_0_ such that

(8) $$\begin{array}{c} \mathbb{P}\left\{\tau_{\epsilon }\leq T\right\}k\;\text{for all }\epsilon \geq \epsilon_{1}. \end{array}$$

For *t* ≥ *τ*_*ϵ*_ and each *ϵ*,

(9) $$\begin{array}{c} \text{d}\left(S+I+R\right)=\left({\left(\hat{A}\right)}^{1-p}{\left(\overset{ˇ}{A}\right)}^{p}-{\left(\hat{\mu }\right)}^{1-p}{\left(\overset{ˇ}{\mu }\right)}^{p}\left(S+I+R\right)-{\left(\hat{r}\right)}^{1-p}{\left(\overset{ˇ}{r}\right)}^{p}I\right)\text{d}t\\ \leq \left({\left(\hat{A}\right)}^{1-p}{\left(\overset{ˇ}{A}\right)}^{p}-{\left(\hat{\mu }\right)}^{1-p}{\left(\overset{ˇ}{\mu }\right)}^{p}\left(S+I+R\right)\right)\text{d}t. \end{array}$$

Then,

(10) $$\begin{array}{c} S\left(t\right)+I\left(t\right)+R\left(t\right)\leq \frac{{\left(\hat{A}\right)}^{1-p}{\left(\overset{ˇ}{A}\right)}^{p}}{{\left(\hat{\mu }\right)}^{1-p}{\left(\overset{ˇ}{\mu }\right)}^{p}}+e^{-{\left(\hat{\mu }\right)}^{1-p}{\left(\overset{ˇ}{\mu }\right)}^{p}t}\left(S_{0}+I_{0}+R_{0}-\frac{{\left(\hat{A}\right)}^{1-p}{\left(\overset{ˇ}{A}\right)}^{p}}{{\left(\hat{\mu }\right)}^{1-p}{\left(\overset{ˇ}{\mu }\right)}^{p}}\right)\\ \leq \left\{\begin{array}{c} \frac{{\left(\hat{A}\right)}^{1-p}{\left(\overset{ˇ}{A}\right)}^{p}}{{\left(\hat{\mu }\right)}^{1-p}{\left(\overset{ˇ}{\mu }\right)}^{p}},\;\text{if }S_{0}+I_{0}+R_{0}\leq \frac{{\left(\hat{A}\right)}^{1-p}{\left(\overset{ˇ}{A}\right)}^{p}}{{\left(\hat{\mu }\right)}^{1-p}{\left(\overset{ˇ}{\mu }\right)}^{p}}\\ S_{0}+I_{0}+R_{0},\;\text{if }S_{0}+I_{0}+R_{0}>\frac{{\left(\hat{A}\right)}^{1-p}{\left(\overset{ˇ}{A}\right)}^{p}}{{\left(\hat{\mu }\right)}^{1-p}{\left(\overset{ˇ}{\mu }\right)}^{p}} \end{array}\right.\\ ≔C. \end{array}$$

Define the following Lyapunov *C*^2^ function *V* : *ℝ*_+_^3^⟶*ℝ*_+_ by

(11) $$\begin{array}{c} V\left(S,I,R\right)=\left(S-1-\ln\;S\right)+\left(I-1-\ln\;I\right)+\left(R-1-\ln\;R\right). \end{array}$$

Obviously, this function is nonnegative which can be seen from *x* − 1 − ln *x* > 0 for *x* > 0.

For 0 ≤ *t* ≤ *τ*_*ϵ*_∧*T*, using Itô's formula, we obtain that

(12) $$\begin{array}{c} \text{d}V\left(S,I,R\right)=ℒV\left(S,I,R\right)\text{d}t-{\left(\hat{\sigma }\right)}^{1-p}{\left(\overset{ˇ}{\sigma }\right)}^{p}S\text{d}W\left(t\right)+{\left(\hat{\sigma }\right)}^{1-p}{\left(\overset{ˇ}{\sigma }\right)}^{p}I\text{d}W\left(t\right)\\ -\int_{Z}\left(\eta \left(u\right)SI+\text{ln}\left(1-\eta \left(u\right)I\right)\right)\tilde{N}\left(\text{d}t,\text{d}u\right)\\ +\int_{Z}\left(\eta \left(u\right)SI-\text{ln}\left(1+\eta \left(u\right)S\right)\right)\tilde{N}\left(\text{d}t,\text{d}u\right), \end{array}$$

where *ℒ* is the differential operator, and

(13) $$\begin{array}{c} ℒV\left(S,I,R\right)=\left(1-\frac{1}{S}\right)\left({\left(\hat{A}\right)}^{1-p}{\left(\overset{ˇ}{A}\right)}^{p}-{\left(\hat{\beta }\right)}^{1-p}{\left(\overset{ˇ}{\beta }\right)}^{p}SI-\left({\left(\hat{\mu }\right)}^{1-p}{\left(\overset{ˇ}{\mu }\right)}^{p}+{\left(\hat{\theta }\right)}^{1-p}{\left(\overset{ˇ}{\theta }\right)}^{p}\right)S\right)\\ +\left(1-\frac{1}{I}\right)\left({\left(\hat{\beta }\right)}^{1-p}{\left(\overset{ˇ}{\beta }\right)}^{p}SI-\left({\left(\hat{\mu }\right)}^{1-p}{\left(\overset{ˇ}{\mu }\right)}^{p}+{\left(\hat{\delta }\right)}^{1-p}{\left(\overset{ˇ}{\delta }\right)}^{p}+{\left(\hat{r}\right)}^{1-p}{\left(\overset{ˇ}{r}\right)}^{p}\right)I\right)\\ +\left(1-\frac{1}{R}\right)\left({\left(\hat{\delta }\right)}^{1-p}{\left(\overset{ˇ}{\delta }\right)}^{p}I+{\left(\hat{\theta }\right)}^{1-p}{\left(\overset{ˇ}{\theta }\right)}^{p}S-{\left(\hat{\mu }\right)}^{1-p}{\left(\overset{ˇ}{\mu }\right)}^{p}R\right)\\ +\frac{1}{2}{\left(\hat{\sigma }\right)}^{2-2p}{\left(\overset{ˇ}{\sigma }\right)}^{2p}I^{2}+\frac{1}{2}{\left(\hat{\sigma }\right)}^{2-2p}{\left(\overset{ˇ}{\sigma }\right)}^{2p}S^{2}\\ -\int_{Z}\left(\text{ln}\left(1-\eta \left(u\right)I\right)+\eta \left(u\right)I\right)\nu \left(\text{d}u\right)-\int_{Z}\left(\text{ln}\left(1+\eta \left(u\right)S\right)-\eta \left(u\right)S\right)\nu \left(\text{d}u\right)\\ \leq {\left(\hat{A}\right)}^{1-p}{\left(\overset{ˇ}{A}\right)}^{p}+{\left(\hat{\beta }\right)}^{1-p}{\left(\overset{ˇ}{\beta }\right)}^{p}C+{\left(\hat{\theta }\right)}^{1-p}{\left(\overset{ˇ}{\theta }\right)}^{p}+{\left(\hat{\sigma }\right)}^{2-2p}{\left(\overset{ˇ}{\sigma }\right)}^{2p}C^{2}+{\left(\hat{r}\right)}^{1-p}{\left(\overset{ˇ}{r}\right)}^{p}\\ +3{\left(\hat{\mu }\right)}^{1-p}{\left(\overset{ˇ}{\mu }\right)}^{p}+{\left(\hat{\delta }\right)}^{1-p}{\left(\overset{ˇ}{\delta }\right)}^{p}+\int_{Z}H_{1}\nu \left(\text{d}u\right)+\int_{Z}H_{2}\nu \left(\text{d}u\right), \end{array}$$

where

(14) $$\begin{array}{c} H_{1}=-\ln\left(1-\eta \left(u\right)I\right)-\eta \left(u\right)I,\\ H_{2}=-\ln\left(1+\eta \left(u\right)S\right)+\eta \left(u\right)S. \end{array}$$

By assumption 1, we have 1 − *η*(*u*)*I* > 0. In addition, by Taylor–Lagrange's formula, we show that

(15) $$\begin{array}{c} H_{1}=\eta \left(u\right)I-\eta \left(u\right)I+\frac{\eta^{2}\left(u\right)I^{2}}{2{\left(1-\kappa \eta \left(u\right)I\right)}^{2}}\leq \frac{\Gamma^{2}}{2{\left(1-\Gamma \right)}^{2}},\;\kappa \in \left(0,1\right). \end{array}$$

Similarly, we get

(16) $$\begin{array}{c} H_{2}=-\eta \left(u\right)S+\eta \left(u\right)S+\frac{\eta^{2}\left(u\right)S^{2}}{2{\left(1+\kappa \eta \left(u\right)S\right)}^{2}}\leq \frac{\Gamma^{2}}{2{\left(1-\Gamma \right)}^{2}},\;\kappa \in \left(0,1\right). \end{array}$$

Therefore,

(17) ℒVS,I,R≤A^1−pAˇp+β^1−pβˇpC+θ^1−pθˇp+σ^2−2pσˇ2pC2+r^1−prˇp+3μ^1−pμˇp+β^1−pβ^p+δ^1−pδ^p+θ^1−pθˇp+Γ21−Γ2νZ≔C˜,

where $\tilde{C}$ is a positive constant. Integrating both sides of (12) from 0 to *τ*_*ϵ*_∧*T*, and taking expectation, we get

(18) $$\begin{array}{c} EV\left(S\left(\tau_{\epsilon }\wedge T\right),I\left(\tau_{\epsilon }\wedge T\right),R\left(\tau_{\epsilon }\wedge T\right)\right)\leq V\left(S_{0},I_{0},R_{0}\right)+\tilde{C}T. \end{array}$$

Setting *Ω*_*ϵ*_={*τ*_*ϵ*_ ≤ *T*} for *ϵ* ≥ *ϵ*_0_ and by (8), we have *ℙ*(*Ω*_*ϵ*_) ≥ *k*. For *ω* ∈ *Ω*_*ϵ*_, there is some component of *S*(*τ*_*ϵ*_), *I*(*τ*_*ϵ*_), and *R*(*τ*_*ϵ*_) equals either *ϵ* or 1/*ϵ*. Hence, *𝒱*(*S*(*τ*_*ϵ*_), *I*(*τ*_*ϵ*_), *R*(*τ*_*ϵ*_)) is not less than *ϵ* − 1 − ln*ϵ* or (1/*ϵ*) − 1 − ln(1/*ϵ*). Consequently,

(19) $$\begin{array}{c} V\left(S\left(0\right),I\left(0\right),R\left(0\right)\right)+\tilde{C}T\geq E\left(1_{\Omega_{\epsilon }}V\left(S\left(\tau_{\epsilon },\omega \right),I\left(\tau_{\epsilon },\omega \right),R\left(\tau_{\epsilon },\omega \right)\right)\right)\\ \geq k\left(\left(\epsilon -1-\text{ln }\epsilon \right)\wedge \left(\frac{1}{\epsilon }-1-\text{ln}\frac{1}{\epsilon }\right)\right). \end{array}$$

Extending *ϵ* to *∞* leads to the contradiction. Thus, *τ*_*∞*_=*∞* a.s. which completes the proof of the theorem.

Remark 2.5. From mathematical and biological considerations, we can study the disease dynamics of the model (5) in the following bounded set:

(20) $$\begin{array}{c} \Delta =\left\{\left(S,I,R\right)\in {\mathbb{R}}_{+}^{3}:\;S+I+R\leq \frac{{\left(\hat{A}\right)}^{1-p}{\left(\overset{ˇ}{A}\right)}^{p}}{{\left(\hat{\mu }\right)}^{1-p}{\left(\overset{ˇ}{\mu }\right)}^{p}}\text{ a}.\text{s}.\right\}. \end{array}$$

Therefore, the region Δ is almost surely positively invariant set by system (5).

### 2.3. Existence and Uniqueness of a Stationary Distribution to System (5)

Our aim in this subsection is to give the appropriate condition for the SDE model (5) which has a unique ergodic stationary distribution. To this end, we introduce the following lemma known as mutually exclusive possibilities. It was proved by Stettner [1].

**Lemma 1 (see [1]).**

Let *X*(*t*) ∈ *ℝ*^*n*^ be a stochastic Feller process, then either an ergodic probability measure exists, or

(21) $$\begin{array}{c} \underset{t⟶\infty }{\lim}\underset{\nu }{\sup}\frac{1}{t}\int_{0}^{t}\mathbb{P}\int \left(u,X_{0},\Sigma \right)\nu \left(\text{d}x\right)\text{d}u=0,\;\text{for any compact set }\Sigma \in {\mathbb{R}}^{n}, \end{array}$$

where the supremum is taken over all initial distributions *ν* on *R*^*d*^ and *ℙ*(*t*, *X*_0_, Σ) is the probability for *X*(*t*) ∈ Σ with *X*(0)=*X*_0_ ∈ *ℝ*^*n*^.

For convenience, we introduce the following notation. Let

(22) $$\begin{array}{c} {ℛ}_{0}^{s}=\frac{1}{\left({\left(\hat{\mu }\right)}^{1-p}{\left(\overset{ˇ}{\mu }\right)}^{p}+{\left(\hat{\delta }\right)}^{1-p}{\left(\overset{ˇ}{\delta }\right)}^{p}+{\left(\hat{r}\right)}^{1-p}{\left(\overset{ˇ}{r}\right)}^{p}\right)}\left(\frac{{\left(\hat{\beta }\hat{A}\right)}^{1-p}{\left(\overset{ˇ}{\beta }\overset{ˇ}{A}\right)}^{p}}{{\left(\hat{\mu }\right)}^{1-p}{\left(\overset{ˇ}{\mu }\right)}^{p}+{\left(\hat{\theta }\right)}^{1-p}{\left(\overset{ˇ}{\theta }\right)}^{p}}-\frac{{\tilde{\sigma }}^{2}{\left(\hat{A}\right)}^{2-2p}{\left(\overset{ˇ}{A}\right)}^{2p}}{2{\left(\hat{\mu }\right)}^{2-2p}{\left(\overset{ˇ}{\mu }\right)}^{2p}}\right), \end{array}$$

where ${\tilde{\sigma }}^{2}={\left(\hat{\sigma }\right)}^{2-2p}{\left(\overset{ˇ}{\sigma }\right)}^{2p}+\int_{Z}\left(\eta^{2}\left(u\right)/{\left(1-\Gamma \right)}^{2}\right)\nu \left(\text{d}u\right)$.

For the ergodicity of system (5), we have the following result.

**Theorem 3 .**

If *ℛ*_0_^*s*^ > 1, the stochastic system (5) admits a unique stationary distribution and it has the ergodic property for any initial value (*S*_0_, *I*_0_, *R*_0_) ∈ Δ.

**Proof**

The following proof is divided into three steps:

  Step I. Similar to the proof of Lemma 3.2 in [24] or Theorem 2.5 in [25], we briefly verify the Feller property of the SDE model (5). The main purpose of the next steps is to prove that (21) is impossible.
  Step II. Define

(23) $$\begin{array}{c} W=\ln\;I+\frac{{\left(\hat{\beta }\right)}^{1-p}{\left(\overset{ˇ}{\beta }\right)}^{p}}{\left({\left(\hat{\mu }\right)}^{1-p}{\left(\overset{ˇ}{\mu }\right)}^{p}+{\left(\hat{\theta }\right)}^{1-p}{\left(\overset{ˇ}{\theta }\right)}^{p}\right)}S. \end{array}$$

  Applying Itô's formula gives

(24) $$\begin{array}{c} \text{d}W\left(t\right)=\left({\left(\hat{\beta }\right)}^{1-p}{\left(\overset{ˇ}{\beta }\right)}^{p}S\left(t\right)-\left({\left(\hat{\mu }\right)}^{1-p}\right.{\left(\overset{ˇ}{\mu }\right)}^{p}+{\left(\hat{\delta }\right)}^{1-p}{\left(\overset{ˇ}{\delta }\right)}^{p}+{\left(\hat{r}\right)}^{1-p}{\left(\overset{ˇ}{r}\right)}^{p}\right)\\ -\frac{1}{2}{\left(\hat{\sigma }\right)}^{2-2p}{\left(\overset{ˇ}{\sigma }\right)}^{2p}S^{2}\left(t^{-}\right)+\int_{Z}\left(\text{ln}\left(1+\eta \left(u\right)S\left(t^{-}\right)\right)-\eta \left(u\right)S\left(t^{-}\right)\nu \left(\text{d}u\right)\right)\text{d}t\\ +{\left(\hat{\sigma }\right)}^{1-p}{\left(\overset{ˇ}{\sigma }\right)}^{p}S\left(t^{-}\right)\text{d}W\left(t\right)+\int_{Z}\text{ln}\left(1+\eta \left(u\right)S\left(t^{-}\right)\right)\tilde{N}\left(\text{d}t,\text{d}u\right)\\ +\frac{{\left(\hat{\beta }\hat{A}\right)}^{1-p}{\left(\overset{ˇ}{\beta }\overset{ˇ}{A}\right)}^{p}}{\left({\left(\hat{\mu }\right)}^{1-p}{\left(\overset{ˇ}{\mu }\right)}^{p}+{\left(\hat{\theta }\right)}^{1-p}{\left(\overset{ˇ}{\theta }\right)}^{p}\right)}\text{d}t-\frac{{\left(\hat{\beta }\right)}^{2-2p}{\left(\overset{ˇ}{\beta }\right)}^{2p}}{\left({\left(\hat{\mu }\right)}^{1-p}{\left(\overset{ˇ}{\mu }\right)}^{p}+{\left(\hat{\theta }\right)}^{1-p}{\left(\overset{ˇ}{\theta }\right)}^{p}\right)}S\left(t\right)I\left(t\right)\text{d}t\\ -\frac{{\left(\hat{\beta }\right)}^{1-p}{\left(\overset{ˇ}{\beta }\right)}^{p}}{\left({\left(\hat{\mu }\right)}^{1-p}{\left(\overset{ˇ}{\mu }\right)}^{p}+{\left(\hat{\theta }\right)}^{1-p}{\left(\overset{ˇ}{\theta }\right)}^{p}\right)}\left({\left(\hat{\mu }\right)}^{1-p}{\left(\overset{ˇ}{\mu }\right)}^{p}+{\left(\hat{\theta }\right)}^{1-p}{\left(\overset{ˇ}{\theta }\right)}^{p}\right)S\left(t\right)\text{d}t\\ -\frac{{\left(\hat{\beta }\right)}^{1-p}{\left(\overset{ˇ}{\beta }\right)}^{p}}{\left({\left(\hat{\mu }\right)}^{1-p}{\left(\overset{ˇ}{\mu }\right)}^{p}+{\left(\hat{\theta }\right)}^{1-p}{\left(\overset{ˇ}{\theta }\right)}^{p}\right)}{\left(\hat{\sigma }\right)}^{1-p}{\left(\overset{ˇ}{\sigma }\right)}^{p}S\left(t^{-}\right)I\left(t^{-}\right)\text{d}W\left(t\right)\\ -\frac{{\left(\hat{\beta }\right)}^{1-p}{\left(\overset{ˇ}{\beta }\right)}^{p}}{\left({\left(\hat{\mu }\right)}^{1-p}{\left(\overset{ˇ}{\mu }\right)}^{p}+{\left(\hat{\theta }\right)}^{1-p}{\left(\overset{ˇ}{\theta }\right)}^{p}\right)}\int_{Z}\eta \left(u\right)S\left(t^{-}\right)I\left(t^{-}\right)\tilde{N}\left(\text{d}t,\text{d}u\right). \end{array}$$

  Noting that $0<S<\left({\left(\hat{A}\right)}^{1-p}{\left(\overset{ˇ}{A}\right)}^{p}/{\left(\hat{\mu }\right)}^{1-p}{\left(\overset{ˇ}{\mu }\right)}^{p}\right)$, the equality (24) can be rewritten as follows:

(25) $$\begin{array}{c} \text{d}W\left(t\right)\geq \left(\frac{{\left(\hat{\beta }\hat{A}\right)}^{1-p}{\left(\overset{ˇ}{\beta }\hat{A}\right)}^{p}}{\left({\left(\hat{\mu }\right)}^{1-p}{\left(\overset{ˇ}{\mu }\right)}^{p}+{\left(\hat{\theta }\right)}^{1-p}{\left(\overset{ˇ}{\theta }\right)}^{p}\right)}-\left({\left(\hat{\mu }\right)}^{1-p}{\left(\overset{ˇ}{\mu }\right)}^{p}+{\left(\hat{\delta }\right)}^{1-p}{\left(\overset{ˇ}{\delta }\right)}^{p}+{\left(\hat{r}\right)}^{1-p}{\left(\overset{ˇ}{r}\right)}^{p}\right)-\frac{{\left(\hat{\sigma }\hat{A}\right)}^{2-2p}{\left(\overset{ˇ}{\sigma }\hat{A}\right)}^{2p}}{2{\left(\hat{\mu }\right)}^{2-2p}{\left(\overset{ˇ}{\mu }\right)}^{2p}}-\frac{{\left(\hat{\beta }\right)}^{2-2p}{\left(\overset{ˇ}{\beta }\right)}^{2p}}{\left({\left(\hat{\mu }\right)}^{1-p}{\left(\overset{ˇ}{\mu }\right)}^{p}+{\left(\hat{\theta }\right)}^{1-p}{\left(\overset{ˇ}{\theta }\right)}^{p}\right)}S\left(t\right)I\left(t\right)\right)\text{d}t\\ +\int_{Z}\left(\text{ln}\left(1+\eta \left(u\right)S\left(t^{-}\right)\right)-\eta \left(u\right)S\left(t^{-}\right)\right)\nu \left(\text{d}u\right)\text{d}t\\ +{\left(\hat{\sigma }\right)}^{1-p}{\left(\overset{ˇ}{\sigma }\right)}^{p}S\left(t^{-}\right)\text{d}W\left(t\right)+\int_{Z}\text{ln}\left(1+\eta \left(u\right)S\left(t^{-}\right)\right)\tilde{N}\left(\text{d}t,\text{d}u\right)\\ -\frac{{\left(\hat{\beta }\right)}^{1-p}{\left(\overset{ˇ}{\beta }\right)}^{p}}{\left({\left(\hat{\mu }\right)}^{1-p}{\left(\overset{ˇ}{\mu }\right)}^{p}+{\left(\hat{\theta }\right)}^{1-p}{\left(\overset{ˇ}{\theta }\right)}^{p}\right)}{\left(\hat{\sigma }\right)}^{1-p}{\left(\overset{ˇ}{\sigma }\right)}^{p}S\left(t^{-}\right)I\left(t^{-}\right)\text{d}W\left(t\right)\\ -\frac{{\left(\hat{\beta }\right)}^{1-p}{\left(\overset{ˇ}{\beta }\right)}^{p}}{\left({\left(\hat{\mu }\right)}^{1-p}{\left(\overset{ˇ}{\mu }\right)}^{p}+{\left(\hat{\theta }\right)}^{1-p}{\left(\overset{ˇ}{\theta }\right)}^{p}\right)}\int_{Z}\eta \left(u\right)S\left(t^{-}\right)I\left(t^{-}\right)\tilde{N}\left(\text{d}t,\text{d}u\right). \end{array}$$

Integrating the inequality (25) from 0 to *t* leads to
  (26) $$\begin{array}{c} W\left(t\right)-W\left(0\right)\geq \frac{{\left(\hat{\beta }\hat{A}\right)}^{1-p}{\left(\overset{ˇ}{\beta }\overset{ˇ}{A}\right)}^{p}}{\left({\left(\hat{\mu }\right)}^{1-p}{\left(\overset{ˇ}{\mu }\right)}^{p}+{\left(\hat{\theta }\right)}^{1-p}{\left(\overset{ˇ}{\theta }\right)}^{p}\right)}-\left({\left(\hat{\mu }\right)}^{1-p}{\left(\overset{ˇ}{\mu }\right)}^{p}+{\left(\hat{\delta }\right)}^{1-p}{\left(\overset{ˇ}{\delta }\right)}^{p}+{\left(\hat{r}\right)}^{1-p}{\left(\overset{ˇ}{r}\right)}^{p}\right)\\ -\frac{{\left(\hat{\sigma }\hat{A}\right)}^{2-2p}{\left(\overset{ˇ}{\sigma }\hat{A}\right)}^{2p}}{2{\left(\hat{\mu }\right)}^{2-2p}{\left(\overset{ˇ}{\mu }\right)}^{2p}}-\frac{{\left(\hat{\beta }\right)}^{2-2p}{\left(\overset{ˇ}{\beta }\right)}^{2p}}{\left({\left(\hat{\mu }\right)}^{1-p}{\left(\overset{ˇ}{\mu }\right)}^{p}+{\left(\hat{\theta }\right)}^{1-p}{\left(\overset{ˇ}{\theta }\right)}^{p}\right)}\int_{0}^{t}S\left(s\right)I\left(s\right)\text{d}s\\ +\int_{0}^{t}\int_{Z}\left(\text{ln}\left(1+\eta \left(u\right)S\left(s^{-}\right)\right)-\eta \left(u\right)S\left(s^{-}\right)\right)\nu \left(\text{d}u\right)\text{d}s\\ +K_{1}\left(t\right)+K_{2}\left(t\right)+K_{3}\left(t\right)+K_{4}\left(t\right), \end{array}$$

where
  (27) $$\begin{array}{c} K_{1}\left(t\right)=\int_{0}^{t}{\left(\hat{\sigma }\right)}^{1-p}{\left(\overset{ˇ}{\sigma }\right)}^{p}S\left(s^{-}\right)\text{d}W\left(s\right),\\ K_{2}\left(t\right)=\frac{-{\left(\hat{\beta }\right)}^{1-p}{\left(\overset{ˇ}{\beta }\right)}^{p}{\left(\hat{\sigma }\right)}^{1-p}{\left(\overset{ˇ}{\sigma }\right)}^{p}}{\left({\left(\hat{\mu }\right)}^{1-p}{\left(\overset{ˇ}{\mu }\right)}^{p}+{\left(\hat{\theta }\right)}^{1-p}{\left(\overset{ˇ}{\theta }\right)}^{p}\right)}\int_{0}^{t}S\left(s^{-}\right)I\left(s^{-}\right)\text{d}W\left(s\right),\\ K_{3}\left(t\right)=\int_{0}^{t}\int_{Z}\text{ln}\left(1+\eta \left(u\right)S\left(s^{-}\right)\right)\tilde{N}\left(\text{d}s,\text{d}u\right),\\ K_{4}\left(t\right)=\frac{-{\left(\hat{\beta }\right)}^{1-p}{\left(\overset{ˇ}{\beta }\right)}^{p}}{\left({\left(\hat{\mu }\right)}^{1-p}{\left(\overset{ˇ}{\mu }\right)}^{p}+{\left(\hat{\theta }\right)}^{1-p}{\left(\overset{ˇ}{\theta }\right)}^{p}\right)}\int_{0}^{t}\int_{Z}\eta \left(u\right)S\left(s^{-}\right)I\left(s^{-}\right)\tilde{N}\left(\text{d}s,\text{d}u\right). \end{array}$$

The quadratic variation of *K*_1_ is defined by ${\langle K_{1},K_{1}\rangle }_{t}=\int_{0}^{t}{\left(\hat{\sigma }\right)}^{2-2p}{\left(\overset{ˇ}{\sigma }\right)}^{2p}S^{2}\left(s\right)\text{d}s$. Therefore, we get
  (28) $$\begin{array}{c} \underset{t⟶\infty }{\text{lim sup}}\frac{{\langle K_{1},K_{1}\rangle }_{t}}{t}={\left(\hat{\sigma }\right)}^{2-2p}{\left(\overset{ˇ}{\sigma }\right)}^{2p}\underset{t⟶\infty }{\text{lim sup}}\frac{1}{t}\int_{0}^{t}S^{2}\left(s^{-}\right)\text{d}s\\ \leq {\left(\hat{\sigma }\right)}^{2-2p}{\left(\overset{ˇ}{\sigma }\right)}^{2p}\frac{{\left(\hat{A}\right)}^{2-2p}{\left(\overset{ˇ}{A}\right)}^{2p}}{{\left(\hat{\mu }\right)}^{2-2p}{\left(\overset{ˇ}{\mu }\right)}^{2p}}<\infty \;\text{a}.\text{s}. \end{array}$$
Similarly, we have
  (29) $$\begin{array}{c} \underset{t⟶\infty }{\text{lim sup}}\frac{{\langle K_{2},K_{2}\rangle }_{t}}{t}=\frac{{\left(\hat{\beta }\right)}^{2-2p}{\left(\overset{ˇ}{\beta }\right)}^{2p}{\left(\hat{\sigma }\right)}^{2-2p}{\left(\overset{ˇ}{\sigma }\right)}^{2p}}{{\left({\left(\hat{\mu }\right)}^{1-p}{\left(\overset{ˇ}{\mu }\right)}^{p}+{\left(\hat{\theta }\right)}^{1-p}{\left(\overset{ˇ}{\theta }\right)}^{p}\right)}^{2}}\underset{t⟶\infty }{\text{lim sup}}\frac{1}{t}\int_{0}^{t}S^{2}\left(s^{-}\right)I^{2}\left(s^{-}\right)\text{d}s<\infty \;\text{a}.\text{s}. \end{array}$$
By the assumption 1, we deduce that
  (30) $$\begin{array}{c} \ln\left(1-\Gamma \right)\leq \;\ln\left(1+\eta \left(u\right)S\left(s^{-}\right)\right)\leq \;\ln\left(1+\Gamma \right). \end{array}$$

Then
  (31) $$\begin{array}{c} \underset{t⟶\infty }{\text{lim sup}}\frac{{\langle K_{3},K_{3}\rangle }_{t}}{t}=\underset{t⟶\infty }{\text{lim sup}}\frac{1}{3}\int_{0}^{t}\int_{Z}\text{ln}{\left(1+\eta \left(u\right)S\left(s^{-}\right)\right)}^{2}\nu \left(\text{d}u\right)\text{d}s\\ \leq \text{max}\left\{{\left(\text{ln}\left(1+\Gamma \right)\right)}^{2},{\left(\text{ln}\left(1-\Gamma \right)\right)}^{2}\right\}\nu \left(Z\right)<\infty \;\text{a}.\text{s}.,\\ \underset{t⟶\infty }{\text{lim sup}}\frac{{\langle K_{4},K_{4}\rangle }_{t}}{t}\leq \frac{{\left(\hat{\beta }A\right)}^{2-2p}{\left(\overset{ˇ}{\beta A}\right)}^{2p}}{\left({\left(\hat{\mu }\right)}^{1-p}{\left(\overset{ˇ}{\mu }\right)}^{p}+{\left(\hat{\theta }\right)}^{1-p}{\left(\overset{ˇ}{\theta }\right)}^{p}\right){\left({\left(\hat{\mu }\right)}^{1-p}{\left(\overset{ˇ}{\mu }\right)}^{p}\right)}^{2}}\nu \left(Z\right)<\infty \;\text{a}.\text{s}. \end{array}$$

According to the strong law of large numbers for local martingales [26], one can conclude that
  (32) $$\begin{array}{c} \underset{t⟶\infty }{\text{lim}}\frac{1}{t}K_{i}\left(t\right)=0,\;\text{a}.\text{s}.,\;i=1,2,3,4. \end{array}$$

By using (16) and assumption 1, we get
  (33) $$\begin{array}{c} \frac{1}{t}\int_{0}^{t}\int_{Z}\left(\text{ln}\left(1+\eta \left(u\right)S\left(s^{-}\right)\right)-\eta \left(u\right)S\left(s^{-}\right)\right)\nu \left(\text{d}u\right)\text{d}s\geq -\frac{1}{2}\frac{{\left(\hat{A}\right)}^{2-2p}{\left(\overset{ˇ}{A}\right)}^{2p}}{{\left(\hat{\mu }\right)}^{2-2p}{\left(\overset{ˇ}{\mu }\right)}^{2p}}\int_{Z}\frac{\eta^{2}\left(u\right)}{{\left(1-\Gamma \right)}^{2}}\nu \left(\text{d}u\right). \end{array}$$
Let
  (34) $$\begin{array}{c} {\tilde{\sigma }}^{2}={\left(\hat{\sigma }\right)}^{2-2p}{\left(\overset{ˇ}{\sigma }\right)}^{2p}+\int_{Z}\frac{\eta^{2}\left(u\right)}{{\left(1-\Gamma \right)}^{2}}\nu \left(\text{d}u\right). \end{array}$$
Therefore,
  (35) $$\begin{array}{c} \underset{t⟶\infty }{\text{lim inf}}\frac{1}{t}\int_{0}^{t}{\left(\hat{\beta }\right)}^{1-p}{\left(\overset{ˇ}{\beta }\right)}^{p}S\left(s\right)I\left(s\right)\text{d}s\geq \frac{\left({\left(\hat{\mu }\right)}^{1-p}{\left(\overset{ˇ}{\mu }\right)}^{p}+{\left(\hat{\theta }\right)}^{1-p}{\left(\overset{ˇ}{\theta }\right)}^{p}\right)}{{\left(\hat{\beta }\right)}^{1-p}{\left(\overset{ˇ}{\beta }\right)}^{p}}\left(\frac{{\left(\hat{\beta }\hat{A}\right)}^{1-p}{\left(\overset{ˇ}{\beta }\overset{ˇ}{A}\right)}^{p}}{\left({\left(\hat{\mu }\right)}^{1-p}{\left(\overset{ˇ}{\mu }\right)}^{p}+{\left(\hat{\theta }\right)}^{1-p}{\left(\overset{ˇ}{\theta }\right)}^{p}\right)}-\left({\left(\hat{\mu }\right)}^{1-p}{\left(\overset{ˇ}{\mu }\right)}^{p}+{\left(\hat{\delta }\right)}^{1-p}{\left(\overset{ˇ}{\delta }\right)}^{p}+{\left(\hat{r}\right)}^{1-p}{\left(\overset{ˇ}{r}\right)}^{p}\right)-{\tilde{\sigma }}^{2}\frac{{\left(\hat{A}\right)}^{2-2p}{\left(\hat{A}\right)}^{2p}}{2\left({\left(\hat{\mu }\right)}^{2-2p}{\left(\overset{ˇ}{\mu }\right)}^{2p}\right)}\right). \end{array}$$
Thus, we can derive that
  (36) $$\begin{array}{c} \underset{t⟶\infty }{\text{lim inf}}\frac{1}{t}\int_{0}^{t}{\left(\hat{\beta }\right)}^{1-p}{\left(\overset{ˇ}{\beta }\right)}^{p}S\left(s\right)I\left(s\right)\text{d}s\geq \frac{\left({\left(\hat{\mu }\right)}^{1-p}{\left(\overset{ˇ}{\mu }\right)}^{p}+{\left(\hat{\theta }\right)}^{1-p}{\left(\overset{ˇ}{\theta }\right)}^{p}\right)}{{\left(\hat{\beta }\right)}^{1-p}{\left(\overset{ˇ}{\beta }\right)}^{p}}\left({\left(\hat{\mu }\right)}^{1-p}{\left(\overset{ˇ}{\mu }\right)}^{p}+{\left(\hat{\delta }\right)}^{1-p}{\left(\overset{ˇ}{\delta }\right)}^{p}+{\left(\hat{r}\right)}^{1-p}{\left(\overset{ˇ}{r}\right)}^{p}\right)\left({ℛ}_{0}^{s}-1\right)>0\;\text{a}.\text{s}. \end{array}$$
Step III. To continue our analysis, we need to set the following subsets:
  (37) $$\begin{array}{c} \Omega_{1}=\left\{\left.\left(S,I,R\right)\in {\mathbb{R}}_{+}^{3}\right|S\geq \epsilon ,\text{ and},I\geq \epsilon \right\},\\ \Omega_{2}=\left\{\left.\left(S,I,R\right)\in {\mathbb{R}}_{+}^{3}\right|S\leq \epsilon \right\},\\ \Omega_{3}=\left\{\left.\left(S,I,R\right)\in {\mathbb{R}}_{+}^{3}\right|I\leq \epsilon \right\}, \end{array}$$
where *ϵ* > 0 is a positive constant to be determined later. It then follows from (36) that
  (38) $$\begin{array}{c} \underset{t⟶+\infty }{\lim\inf}\frac{1}{t}\int_{0}^{t}E\left({\left(\hat{\beta }\right)}^{1-p}{\left(\overset{ˇ}{\beta }\right)}^{p}S\left(u\right)I\left(u\right)1_{\Omega_{1}}\right)\text{d}u\geq \underset{t⟶+\infty }{\lim\inf}\frac{1}{t}\int_{0}^{t}E\left({\left(\hat{\beta }\right)}^{1-p}{\left(\overset{ˇ}{\beta }\right)}^{p}S\left(u\right)I\left(u\right)\right)\text{d}u\\ -\underset{t⟶+\infty }{\lim\sup}\frac{1}{t}\int_{0}^{t}E\left({\left(\hat{\beta }\right)}^{1-p}{\left(\overset{ˇ}{\beta }\right)}^{p}S\left(u\right)I\left(u\right)1_{\Omega_{2}}\right)\text{d}u\\ -\underset{t⟶+\infty }{\lim\sup}\frac{1}{t}\int_{0}^{t}E\left({\left(\hat{\beta }\right)}^{1-p}{\left(\overset{ˇ}{\beta }\right)}^{p}S\left(u\right)I\left(u\right)1_{\Omega_{3}}\right)\text{d}u\\ \geq \frac{\left({\left(\hat{\mu }\right)}^{1-p}{\left(\overset{ˇ}{\mu }\right)}^{p}+{\left(\hat{\theta }\right)}^{1-p}{\left(\overset{ˇ}{\theta }\right)}^{p}\right)}{{\left(\hat{\beta }\right)}^{1-p}{\left(\overset{ˇ}{\beta }\right)}^{p}}\left({\left(\hat{\mu }\right)}^{1-p}{\left(\overset{ˇ}{\mu }\right)}^{p}+{\left(\hat{\delta }\right)}^{1-p}{\left(\overset{ˇ}{\delta }\right)}^{p}+{\left(\hat{r}\right)}^{1-p}{\left(\overset{ˇ}{r}\right)}^{p}\right)\left({ℛ}_{0}^{s}-1\right)\\ -\frac{2{\left(\hat{\beta }\hat{A}\right)}^{1-p}{\left(\overset{ˇ}{\beta }\overset{ˇ}{A}\right)}^{p}\epsilon }{{\left(\hat{\mu }\right)}^{1-p}{\left(\overset{ˇ}{\mu }\right)}^{p}}. \end{array}$$

We can choose

(39) $$\begin{array}{c} \epsilon \leq \frac{\left({\left(\hat{\mu }\right)}^{1-p}{\left(\overset{ˇ}{\mu }\right)}^{p}+{\left(\hat{\theta }\right)}^{1-p}{\left(\overset{ˇ}{\theta }\right)}^{p}\right)\left({\left(\hat{\mu }\right)}^{1-p}{\left(\overset{ˇ}{\mu }\right)}^{p}\right)}{4{\left(\hat{\beta }\right)}^{2-2p}{\left(\overset{ˇ}{\beta }\right)}^{2p}{\left(\hat{A}\right)}^{1-p}{\left(\overset{ˇ}{A}\right)}^{p}}\left({\left(\hat{\mu }\right)}^{1-p}{\left(\overset{ˇ}{\mu }\right)}^{p}+{\left(\hat{\delta }\right)}^{1-p}{\left(\overset{ˇ}{\delta }\right)}^{p}+{\left(\hat{r}\right)}^{1-p}{\left(\overset{ˇ}{r}\right)}^{p}\right)\left({ℛ}_{0}^{s}-1\right), \end{array}$$

then, we obtain

(40) $$\begin{array}{c} \underset{t⟶+\infty }{\lim\inf}\frac{1}{t}\int_{0}^{t}E\left({\left(\hat{\beta }\right)}^{1-p}{\left(\overset{ˇ}{\beta }\right)}^{p}S\left(u\right)I\left(u\right)1_{\Omega_{1}}\right)\text{d}u\geq \frac{\left({\left(\hat{\mu }\right)}^{1-p}{\left(\overset{ˇ}{\mu }\right)}^{p}+{\left(\hat{\theta }\right)}^{1-p}{\left(\overset{ˇ}{\theta }\right)}^{p}\right)}{2{\left(\hat{\beta }\right)}^{1-p}{\left(\overset{ˇ}{\beta }\right)}^{p}}\left({\left(\hat{\mu }\right)}^{1-p}{\left(\overset{ˇ}{\mu }\right)}^{p}+{\left(\hat{\delta }\right)}^{1-p}{\left(\overset{ˇ}{\delta }\right)}^{p}+{\left(\hat{r}\right)}^{1-p}{\left(\overset{ˇ}{r}\right)}^{p}\right)\left({ℛ}_{0}^{s}-1\right)>0\;\text{a}.\text{s}. \end{array}$$

Let *a* and *b* two real numbers greater than 1 such that (1/*a*)+(1/*b*)=1. By utilizing Young inequality *xy* ≤ (*x*^*a*^/*a*)+(*y*^*b*^/*b*) for all *x*, *y* > 0, we get

(41) $$\begin{array}{c} \underset{t⟶+\infty }{\lim\inf}\frac{1}{t}\int_{0}^{t}E\left({\left(\hat{\beta }\right)}^{1-p}{\left(\overset{ˇ}{\beta }\right)}^{p}S\left(u\right)I\left(u\right)1_{\Omega_{1}}\right)\text{d}u\\ \leq \underset{t⟶+\infty }{\lim\inf}\frac{1}{t}\int_{0}^{t}E\left(a^{-1}{\left(\varpi {\left(\hat{\beta }\right)}^{1-p}{\left(\overset{ˇ}{\beta }\right)}^{p}S\left(u\right)I\left(u\right)\right)}^{a}+b^{-1}\varpi^{-b}1_{\Omega_{1}}\right)\text{d}u\\ \leq a^{-1}{\left(\varpi {\left(\hat{\beta }\right)}^{1-p}{\left(\overset{ˇ}{\beta }\right)}^{p}\right)}^{a}{\left(\frac{{\left(\hat{A}\right)}^{1-p}{\left(\overset{ˇ}{A}\right)}^{p}}{{\left(\hat{\mu }\right)}^{1-p}{\left(\overset{ˇ}{\mu }\right)}^{p}}\right)}^{2a}+\underset{t⟶+\infty }{\lim\inf}\frac{1}{t}\int_{0}^{t}E\left(b^{-1}\varpi^{-b}1_{\Omega_{1}}\right)\text{d}u, \end{array}$$

where *ϖ* is a positive constant satisfying

(42) $$\begin{array}{c} \varpi^{a}\leq \frac{a}{4}{\left({\left(\hat{\beta }\right)}^{1-p}{\left(\overset{ˇ}{\beta }\right)}^{p}\right)}^{-\left(a+1\right)}{\left(\frac{{\left(\hat{A}\right)}^{1-p}{\left(\overset{ˇ}{A}\right)}^{p}}{{\left(\hat{\mu }\right)}^{1-p}{\left(\overset{ˇ}{\mu }\right)}^{p}}\right)}^{-2a}\left({\left(\hat{\mu }\right)}^{1-p}{\left(\overset{ˇ}{\mu }\right)}^{p}+{\left(\hat{\theta }\right)}^{1-p}{\left(\overset{ˇ}{\theta }\right)}^{p}\right)\left({\left(\hat{\mu }\right)}^{1-p}{\left(\overset{ˇ}{\mu }\right)}^{p}+{\left(\hat{\delta }\right)}^{1-p}{\left(\overset{ˇ}{\delta }\right)}^{p}+{\left(\hat{r}\right)}^{1-p}{\left(\overset{ˇ}{r}\right)}^{p}\right)\left({ℛ}_{0}^{s}-1\right). \end{array}$$

From (41), we deduce that

(43) $$\begin{array}{c} \underset{t⟶+\infty }{\lim\inf}\frac{1}{t}\int_{0}^{t}E\left(1_{\Omega_{1}}\right)\text{d}u\geq \frac{\left({\left(\hat{\mu }\right)}^{1-p}{\left(\overset{ˇ}{\mu }\right)}^{p}+{\left(\hat{\theta }\right)}^{1-p}{\left(\overset{ˇ}{\theta }\right)}^{p}\right)b\varpi^{b}}{4{\left(\hat{\beta }\right)}^{1-p}{\left(\overset{ˇ}{\beta }\right)}^{p}}\left({\left(\hat{\mu }\right)}^{1-p}{\left(\overset{ˇ}{\mu }\right)}^{p}+{\left(\hat{\delta }\right)}^{1-p}{\left(\overset{ˇ}{\delta }\right)}^{p}+{\left(\hat{r}\right)}^{1-p}{\left(\overset{ˇ}{r}\right)}^{p}\right)\left({ℛ}_{0}^{s}-1\right)>0\;\text{a}.\text{s}. \end{array}$$

Setting

(44) $$\begin{array}{c} \Omega_{4}=\left\{\left(S,I,R\right)\in {\mathbb{R}}_{+}^{3}\left|S\geq \zeta \right.,\text{or},I\geq \zeta \right\},\\ \Sigma =\left\{\left(S,I,R\right)\in {\mathbb{R}}_{+}^{3}\left|\epsilon \leq S\leq \zeta \right.,\text{and},\epsilon \leq I\leq \zeta \right\}, \end{array}$$

where *ζ* > 0 is a positive constant to be explained in the following. By using the Tchebychev inequality, we can observe that

(45) $$\begin{array}{c} E\left(1_{\Omega_{4}}\right)\leq \mathbb{P}\left(S\left(t\right)\geq \zeta \right)+\mathbb{P}\left(I\left(t\right)\geq \zeta \right)\leq \frac{1}{\zeta }E\left(S\left(t\right)+I\left(t\right)\right)\leq \frac{1}{\zeta }\frac{{\left(\hat{A}\right)}^{1-p}{\left(\overset{ˇ}{A}\right)}^{p}}{{\left(\hat{\mu }\right)}^{1-p}{\left(\overset{ˇ}{\mu }\right)}^{p}}. \end{array}$$

Choosing

(46) $$\begin{array}{c} \frac{1}{\zeta }\leq \frac{\left({\left(\hat{\mu }\right)}^{1-p}{\left(\overset{ˇ}{\mu }\right)}^{p}+{\left(\hat{\theta }\right)}^{1-p}{\left(\overset{ˇ}{\theta }\right)}^{p}\right)b\varpi^{b}}{8{\left(\hat{\beta }\hat{A}\right)}^{1-p}{\left(\overset{ˇ}{\beta }\overset{ˇ}{A}\right)}^{p}}\left({\left(\hat{\mu }\right)}^{1-p}{\left(\overset{ˇ}{\mu }\right)}^{p}+{\left(\hat{\delta }\right)}^{1-p}{\left(\overset{ˇ}{\delta }\right)}^{p}+{\left(\hat{r}\right)}^{1-p}{\left(\overset{ˇ}{r}\right)}^{p}\right)\left({ℛ}_{0}^{s}-1\right). \end{array}$$

We thus obtain

(47) $$\begin{array}{c} \underset{t⟶+\infty }{\lim\sup}\frac{1}{t}\int_{0}^{t}E\left(1_{\Omega_{4}}\right)\text{d}u\leq \frac{\left({\left(\hat{\mu }\right)}^{1-p}{\left(\overset{ˇ}{\mu }\right)}^{p}+{\left(\hat{\theta }\right)}^{1-p}{\left(\overset{ˇ}{\theta }\right)}^{p}\right)b\varpi^{b}}{8{\left(\hat{\beta }\right)}^{1-p}{\left(\overset{ˇ}{\beta }\right)}^{p}}\left({\left(\hat{\mu }\right)}^{1-p}{\left(\overset{ˇ}{\mu }\right)}^{p}+{\left(\hat{\delta }\right)}^{1-p}{\left(\overset{ˇ}{\delta }\right)}^{p}+{\left(\hat{r}\right)}^{1-p}{\left(\overset{ˇ}{r}\right)}^{p}\right)\left({ℛ}_{0}^{s}-1\right). \end{array}$$

According to (43), one can derive that

(48) liminft⟶+∞1tE∫0t1Σdu≥liminft⟶+∞1t∫0tE1Ω1du−limsupt⟶+∞1t∫0tE1Ω4du≥μ^1−pμˇp+θ^1−pθˇpbϖb8 ^β1−pβˇpμ^1−pμˇp+δ^1−pδˇp+r^1−prˇpℛ0s−1>0 a.s.

Based on the above analysis, we have determined a compact domain Σ ⊂ *ℝ*_+_^3^ such that

(49) $$\begin{array}{c} \underset{t⟶+\infty }{\lim\inf}\frac{1}{t}\int_{0}^{t}\mathbb{P}\left(u,\left(S_{0},I_{0},R_{0}\right),\Sigma \right)\text{d}u\geq \frac{\left({\left(\hat{\mu }\right)}^{1-p}{\left(\overset{ˇ}{\mu }\right)}^{p}+{\left(\hat{\theta }\right)}^{1-p}{\left(\overset{ˇ}{\theta }\right)}^{p}\right)b\varpi^{b}}{8{\left(\hat{\beta }\right)}^{1-p}{\left(\overset{ˇ}{\beta }\right)}^{p}}\left({\left(\hat{\mu }\right)}^{1-p}{\left(\overset{ˇ}{\mu }\right)}^{p}+{\left(\hat{\delta }\right)}^{1-p}{\left(\overset{ˇ}{\delta }\right)}^{p}+{\left(\hat{r}\right)}^{1-p}{\left(\overset{ˇ}{r}\right)}^{p}\right)\left({ℛ}_{0}^{s}-1\right)>0\;\text{a}.\text{s}. \end{array}$$

Applying similar arguments to those in [24], we show the uniqueness of the ergodic stationary distribution of our model (5), denoted by *π*(·). This completes the proof.

### 2.4. Numerical Simulations

**Figure 1:**
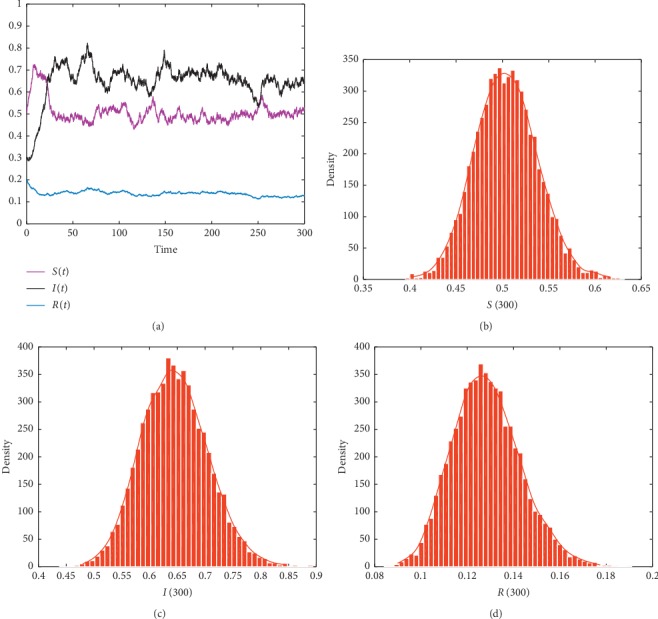
The trajectories and histogram of solution of model (5) with initial value (*S*_0_, *I*_0_, *R*_0_)=(0.5, 0.3, 0.2) and *p*=1.

**Figure 2:**
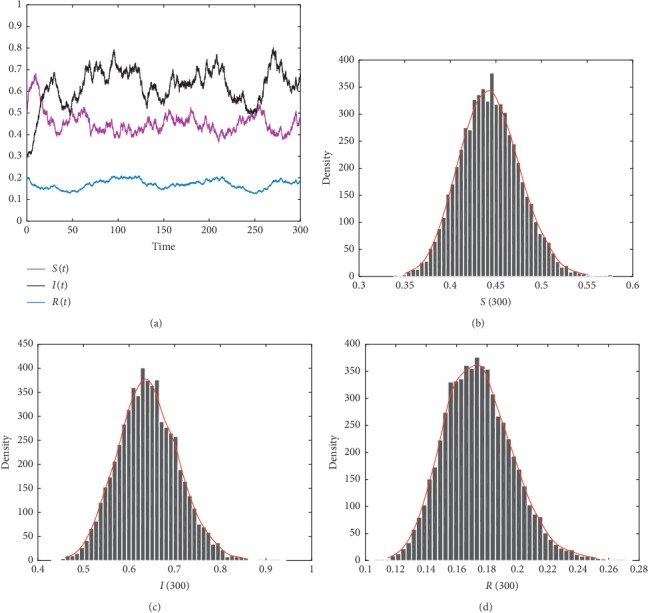
The trajectories and histogram of solution of model (5) with initial value (*S*_0_, *I*_0_, *R*_0_)=(0.5, 0.3, 0.2) and *p*=0.5.

**Figure 3:**
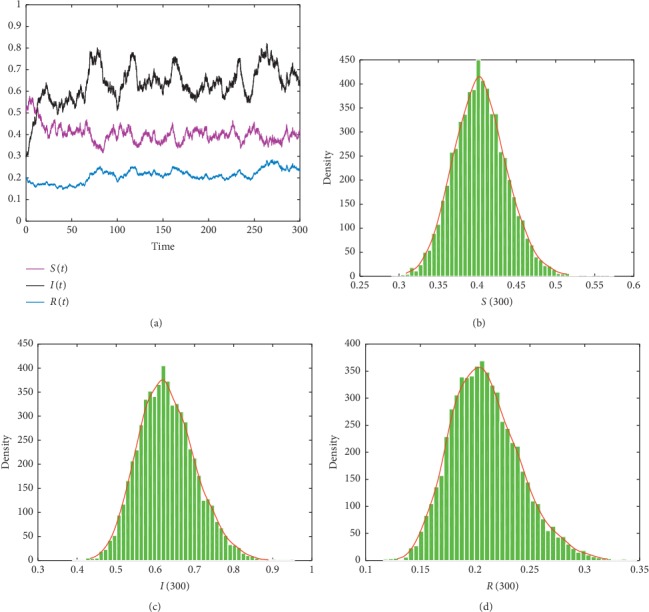
The trajectories and histogram of solution of model (5) with initial value (*S*_0_, *I*_0_, *R*_0_)=(0.5, 0.3, 0.2) and *p*=0.

In this subsection, in order to show different dynamical results of the stochastic model (2) under imprecise parameter values, we present some numerical simulations. We use Milstein's method to simulate the trajectories of the stochastic model (5). The parameters values are given in the following list. For the purpose of showing the effects of imprecise parameters and Lévy noise on Hepatitis B dynamics, we have realized the simulation 10000 times. We assume that *η*(*u*)=0.03, *Z*=(0, *∞*), and *ν*(*Z*)=1. Then, we obtain the following results: noticing that the assumption 1 is always held with parameters' value in Table 2. From Figures 1–3, we show the existence of the unique stationary distributions for *S*(*t*), *I*(*t*), and *R*(*t*) of model (5) at *t*=300, where the smooth curves are the probability density functions of *S*(*t*), *I*(*t*), and *R*(*t*), respectively. It can be obviously observed that the solution of the SDE model (5) persists in the mean. Furthermore, different values of the parameter imprecision *p* can also crucially affect the persistence of Hepatitis B (see Table 3).

## 3. Discussion

In the study of the dynamics of stochastic systems, the existence of an ergodic stationary distribution is one of the most important and significant characteristics. For this purpose, we have used the Feller property and mutually exclusive possibilities lemma to establish the sharp and optimal condition for the existence of the stationary distribution without employing the classical Lyapunov method. To ensure the realistic aspect of our model, we replaced constant parameters in the model (2) by imprecise ones. Based on Theorem 4.2 in [23], for any π-integrable function *g* : *ℝ*_+_⟶*ℝ*,

(50) $$\begin{array}{c} \mathbb{P}\left(\underset{t⟶+\infty }{\lim}\frac{1}{t}\int_{0}^{t}g\left(X\left(s\right)\right)\text{d}s=\int_{{\mathbb{R}}_{+}}g\left(x\right)\pi \left(x\right)\text{d}x\right)=1. \end{array}$$

The ergodic property for HBV means that the stochastic model has a unique stationary distribution which predicts the survival of the infected population in the future. That means the HBV persists for all time regardless of the initial conditions [27]. Furthermore, the ergodic property grants a reason why the integral average of a solution of system (5) converges to a fixed point whilst the system may fluctuate around as time goes by.

## Figures and Tables

**Table 1:** Biological meanings of the parameters in model (1).

Parameters	Interpretation
*A*	The recruitment rate corresponding to births and immigration.
*μ*	The natural mortality rate.
*β*	The transmission rate.
*δ*	The rate of individuals leaving *I* to *R*.
*r*	The disease-related death rate.
*θ*	The successful vaccination rate.

**Table 2:** Parameters' value used in numerical simulations.

Notation	Value	Source	Notation	Value
$\hat{A}$	0.4	[9]	$\overset{ˇ}{A}$	0.6
$\hat{\mu }$	0.09	[9]	$\overset{ˇ}{\mu }$	0.2
$\hat{\beta }$	0.1	[9]	$\overset{ˇ}{\beta }$	0.2
$\hat{\delta }$	0.3	[9]	$\overset{ˇ}{\delta }$	0.5
$\hat{r}$	0.1	[9]	$\overset{ˇ}{r}$	0.3
$\hat{\theta }$	0.2	Assumed	$\overset{ˇ}{\theta }$	0.3
$\hat{\sigma }$	0.08	Assumed	$\overset{ˇ}{\sigma }$	0.1

**Table 3:** Examples of some value of *p* and their numerical illustrations.

Parameter imprecision	Value of *ℛ*_0_^*s*^	Simulation result
*p*=1	1.1192	Figure 1
*p*=0.5	1.5812	Figure 2
*p*=0	2.1816	Figure 3

## Data Availability

The theoretical data used to support the findings of this study are included within the article.
